# Hollow Fiber Membrane Contactors for Post-Combustion Carbon Capture: A Review of Modeling Approaches

**DOI:** 10.3390/membranes10120382

**Published:** 2020-11-30

**Authors:** Joanna R. Rivero, Grigorios Panagakos, Austin Lieber, Katherine Hornbostel

**Affiliations:** 1 Department of Mechanical Engineering and Material Science, University of Pittsburgh, 3700 O’Hara St, Pittsburgh, PA 15213, USA; jrr103@pitt.edu (J.R.R.); arl127@pitt.edu (A.L.); hornbostel@pitt.edu (K.H.); 2 Department of Chemical Engineering, Carnegie Mellon University, 5000 Forbes Ave, Pittsburgh, PA 15213, USA

**Keywords:** hollow fiber membrane contactor modeling, post-combustion carbon capture, carbon capture membrane modeling

## Abstract

Hollow fiber membrane contactors (HFMCs) can effectively separate CO${}_{2}$ from post-combustion flue gas by providing a high contact surface area between the flue gas and a liquid solvent. Accurate models of carbon capture HFMCs are necessary to understand the underlying transport processes and optimize HFMC designs. There are various methods for modeling HFMCs in 1D, 2D, or 3D. These methods include (but are not limited to): resistance-in-series, solution-diffusion, pore flow, Happel’s free surface model, and porous media modeling. This review paper discusses the state-of-the-art methods for modeling carbon capture HFMCs in 1D, 2D, and 3D. State-of-the-art 1D, 2D, and 3D carbon capture HFMC models are then compared in depth, based on their underlying assumptions. Numerical methods are also discussed, along with modeling to scale up HFMCs from the lab scale to the commercial scale.

## 1. Introduction

In 2018, the Intergovernmental Panel on Climate Change issued a report detailing the irreversible impact of a global temperature rise of 1.5 ${}^{\circ }$C [1]. Though many countries are transitioning towards clean energy, a complete infrastructural shift from fossil fuels may take several decades. Carbon capture technology is critical to bridge the gap during this transition. Furthermore, natural gas plants may be necessary long-term to provide cheap baseload power to supplement intermittent renewable sources. Coal-fired and natural gas power plants in the United States are a target market for carbon capture (CC). This is because coal-fired power plants contribute 973 Million metric tons (MMmt) of CO${}_{2}$ to the atmosphere annually [2], and natural gas power plants contribute about 619 MMmt [3]. State-of-the-art methods for power plant CC include (but are not limited to): physical and chemical solvation and adsorption, cryogenic separation, and membrane separation [4,5]. Membrane-based methods offer several distinct advantages, including: minimal energy input, smaller unit footprint, ease of retrofit and replacement, quick response to power plant dynamics, and environmental friendliness.

Most membrane CC technologies flow power plant flue gas on one side and pull a vacuum on the other side to drive CO${}_{2}$ diffusion. These membrane systems often pressurize the flue gas before it reaches the membrane to raise the CO${}_{2}$ partial pressure difference across the membrane, which increases the rate of CO${}_{2}$ diffusion. Pressurizing the flue gas, however, is energy-intensive and raises operating costs. Conventional CC membranes consist of a selective layer on top of a porous support layer to prevent nitrogen and other gaseous species from crossing over with the CO${}_{2}$ [6]. These selective layers, however, raise the membrane cost and lower the flux of CO${}_{2}$ because they add resistance to CO${}_{2}$ transport.

One alternative design to conventional CC membranes is the membrane contactor, which enables gas exchange between two fluid streams [7]. Membrane contactors use a fluid sweep instead of a vacuum to drive CO${}_{2}$ across the membrane. Membrane contactors that use a liquid solvent sweep are particularly effective because the solvent is selective to CO${}_{2}$ over other gas species. This means that the membrane does not need a selective layer to block other gases, which can lower membrane cost and boost CO${}_{2}$ flux.

Many gas-liquid membrane contactors use carbon capture solvents, which react with CO${}_{2}$, driving more CO${}_{2}$ across the membrane. Although energy is required to then strip CO${}_{2}$ from the solvent in a regeneration process, gas-liquid membrane contactors have several competitive advantages over other membrane configurations: no need to pressurize the flue gas (which requires a lot of energy), higher CO${}_{2}$ fluxes, no selective layer, and independent flow regulation [8]. Another advantage for membrane contactors is that the mass transfer resistance from the membrane is minimum in comparison to the gas and liquid mass transfer resistance. However, if membrane wetting occurs, the mass transfer resistance on the membrane increases and result in poor separation performance [8]. Gas-liquid membrane contactors used in post-combustion carbon capture (PCC) are the focus of this review paper.

The membrane in a membrane contactor is generally thin and has a high surface area per unit volume to promote CO${}_{2}$ transfer. Material selection is half of the challenge when designing a CC membrane contactor. The optimal materials for a CC membrane depend on the makeup and flow rate of the feed gas, system operating conditions, and separation requirements [9]. Although membrane contactors do not require a selective layer (because the solvent is already selective to CO${}_{2}$), they often require a thin coating to prevent membrane wetting. The main membrane material must also be designed to withstand prolonged contact with flue gas and with the solvent of choice. Many experimental studies have been conducted to study the performance and lifespan of different membrane material and solvent combinations. Table 1 and Table 2 cover several common solvents and membrane materials used in PCC gas-liquid membrane contactors [10].

It should be noted that both physical and chemical solvents are used in the field of CC. For the physical solvent, there is no chemical reaction as the system is based purely on gas solubility. However, once a chemical solvent is added to the system, there is a chemical reaction on the solvent side. Although this review paper focuses primarily on modeling membrane contactors, other review papers have done a comprehensive job of comparing membrane materials and physical and chemical solvents [8,9,11,12,13]. The reaction modeling for membrane contactors using solvents will not be discussed in this review paper. However, the papers cited in Table 1 provide a good starting point for modeling reaction chemistry for these common solvents.

**Figure 1:**
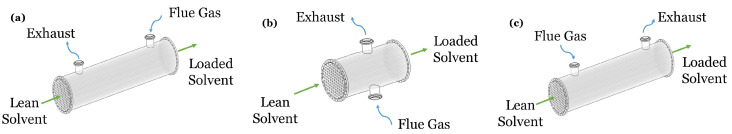
Illustration of flow patterns of a gas-liquid HFMC for post-combustion carbon capture (PCC) (**a**) counter-current flow, (**b**) cross-flow, and (**c**) co-current flow.

Carbon capture membrane contactors come in many configurations: tubular/hollow-fiber [38], spiral wound [39], and flat sheet [40]. A common geometric configuration used in PCC is the hollow fiber membrane contactor (HFMC), and this particular design will be the focus of this review paper. HFMCs consist of many long, narrow, hollow fibers packed into bundles. HFMCs are manufactured by first creating a woven fabric-like bundle using a rotating wheel [41]. The ends of the bundle are then fused shut and undergo centrifugal potting to form a tubesheet. This tubesheet’s ends are then cut to recreate open-ended fibers. The bundle with tubesheet ends is then placed inside a case that has two ports for the permeate fluid and two headers at each end for the inside of the hollow fiber, known as the lumen access port [42]. HFMCs for PCC systems are relatively inexpensive, have a high surface area per unit volume, and are easy to seal, preventing leakage. HFMCs are also relatively easy to model compared to other CO${}_{2}$ separation methods due to their simple geometry and flow configuration. HFMCs are commonly operated in a counter-flow configuration (where the sweep gas or solvent runs in the opposite direction that the exhaust gas flows). However, they can also be operated in co-flow (where sweep runs in the same direction to the exhaust) or in cross-flow (where sweep runs perpendicular to the exhaust), as seen in Figure 1.

A common performance metric for HFMCs is the percentage removal rate, defined as follows [22,43]:

(1) $$\%\;{\mathrm{removal}\;\mathrm{of}\;\mathrm{CO}}_{2}=100\left(1-\frac{C_{CO_{2},outlet}}{C_{CO_{2},inlet}}\right)=100\left(\frac{{\left(\dot{n}\right)}_{inlet}-{\left(\dot{n}\right)}_{outlet}}{{\left(\dot{n}\right)}_{inlet}}\right),$$

where $\dot{n}\left[\frac{mol}{s}\right]$ is the molar flow rate defined as the volumetric flow rate times the concentration, $\dot{v}C_{CO_{2}}$, or, in a more general sense, $\int_{S}uC\;d\vec{S}$. Another common performance metric in CC HFMCs is the effective capture ratio, η, which is a function of interfacial area, $a[m^{2}]$, gas velocity $v_{g}\left[\frac{m}{s}\right]$, fiber length, L[m], and the inverse of the CO${}_{2}$ transfer time, *K* [44]:

(2) $$\eta =1-exp\left(\frac{-KaL}{v_{g}}\right).$$

There are already several review papers on the topic of HFMCs for PCC. An in-depth explanation of gas-liquid HFMCs is offered by Gabelman et al., who address theoretical and design considerations, mass transfer performance and applications [45]. Ji et al. give a more recent theoretical and application-specific review on membrane-based technology, including HFMCs [46]. Optimization methods, experimental approaches, and energetic and economic evaluations have been performed on HFMCs to stress the highest packing density and lowest cost in comparison to other configurations [47]. Other HFMC review papers have focused on scale-up and industrial application of HFMC CO${}_{2}$ capture technology [8,48,49,50]. Some review papers also cover wetting effects and mass transfer correlations in 1D and 2D only [19,44,51].

These state-of-the-art HFMC review papers also focus on experimental work. A major challenge with performing experimental studies in this field is that it is difficult to translate bench-scale results to a commercial system, particularly when experimental studies do not report costs or system configurations [52]. It often takes over a decade to translate lab-scale technology to commercialization, and this process often requires multiple rounds of expensive pilot testing at facilities like the National Carbon Capture Center (NCCC) [53]. Modeling is necessary to expedite this scale-up process and guide experimental design to save time and money. Furthermore, experiments conducted in this field are often tailored to a unique set of membrane materials and operating conditions. This makes it challenging to compare reported experimental results or determine the optimal membrane contactor design. Modeling is needed to bridge this gap between disparate experimental results and determine the optimal design. Modeling and optimization can help minimize the many costs associated with membrane contactor systems (e.g., membrane materials, manufacturing, and operating costs [54]).

This paper offers a comprehensive review of modeling studies to date for gas-liquid HFMCs for PCC. The purposes of this review are: (1) to present the current field of HFMC carbon capture modeling, (2) to compare 1D, 2D, and 3D HFMC modeling approaches, and (3) to identify areas for future research in HFMC modeling. The paper is organized into 1D, 2D, and 3D modeling sections. These sections are followed by a section comparing the 1D, 2D, and 3D modeling approaches. Finally, numerical approaches and modeling to assist scaling up HFMC technology are discussed.

## 2. Fundamental Theory

Before presenting the theory of 1D, 2D, and 3D modeling, this section will briefly cover the governing equations and constitutive laws used to model HFMCs that can be found in related transport textbooks [55]. This is to stress the need of understanding the fundamentals of mass transport for successful modeling.

### 2.1. Constitutive Laws

The constitutive laws are needed to derive the relevant governing equations for a complete mass transfer analysis. These laws provide micro scale analysis (e.g., describe the molecule interaction) for a complete macro scale analysis (e.g., describe the bulk interaction). The constitutive laws are based on Fick’s first law of diffusion (Equation (3)), and the strain-rate relationship (also known as Newton’s law of viscosity):

(3) $$J_{j}=-D_{j}\nabla C_{j},$$

(4) $$\tau =-\mu \left(\nabla v+{\left(\nabla v\right)}^{⊺}\right)+\left(\frac{2}{3}\mu -\kappa \right)\left(\nabla \cdot v\right)\delta .$$

Fick’s first law of diffusion is defined based on the diffusion coefficient, $D[\frac{m^{2}}{s}]$, the concentration, $C[\frac{mol}{m^{3}}]$, and the species, *j*. Newton’s law of viscosity is defined based on the viscous momentum flux tensor, τ[Pa], the velocity vector, $v[\frac{m}{s}]$, the fluid viscosity μ[Pa·s], and the unit tensor, δ. Previous work also assumes monatomic gases at low density, making the dilatational viscosity, κ, equal zero.

In the case heat transfer is being considered, Fourier’s law should be considered to derive the thermal energy equation:

(5) q=−k∇T.

For Equations (3)–(5), the material properties do not have to be constant and can be functions of parameters of the system, such as temperature, concentration, and pressure.

### 2.2. Governing Equations

The governing equations of motion used throughout this analysis are the conservation of mass, linear momentum, convection–diffusion, and energy equations [56,57,58]. The conservation of mass equation, also known as the continuity equation for species, is derived from Fick’s first law of diffusion (Equation (3)).

(6) $$\frac{\partial C_{j}}{\partial t}+\nabla \cdot \left(C_{j}v\right)=r_{j}+f.$$

Equation (6) considers the concentration, $C[\frac{mol}{m^{3}}]$; the velocity vector, $v[\frac{m}{s}]$; the reaction rate, $r_{j}\left[\frac{mol}{m^{3}s}\right]$; and a constitutive parameter, *f*, as a function that is to be determined by the constitutive theory [58]. The equation of motion in terms of the viscous momentum flux (Equation (4)) and the continuity equation (conservation of mass) is shown below:

(7) $$\rho \frac{Dv}{Dt}=\nabla \cdot \tau +\rho b,$$

(8) $$\frac{\partial \rho }{\partial t}+\nabla \cdot \left(\rho v\right)=0,$$

where $\frac{D}{Dt}$ is the substantial time derivative, given by: $\frac{\partial }{\partial t}+\nabla \left(\;\right)v$. b is the body force vector, $v[\frac{m}{s}]$ is the velocity vector, and $\rho [\frac{kg}{m^{3}}]$ is the density. Equation (7) can be derived if Equation (6) is written in a per mass basis and summed over all species *j*.

In the case where non-isothermal conditions are considered, the energy equation, derived from Equation (5), should also be included in the transport analysis [59].

(9) $$\rho \frac{D\epsilon }{Dt}=\tau :L-\nabla \cdot q+r_{rxn}\Delta H_{rxn}M.$$

Equation (9) includes the substantial time derivative, $\frac{D}{Dt}$; the specific internal energy, ϵ; the gradient of the velocity, *L*; the heat flux vector from Equation (5), q; and the total reaction rate $r_{rxn}\left[\frac{mol}{m^{3}s}\right]$, multiplied by the enthaply, $\Delta H_{rxn}\left[\frac{J}{kg}\right]$; and the molecular weight, $M[\frac{kg}{mol}]$.

## 3. One-Dimensional Modeling

**Figure 2:**
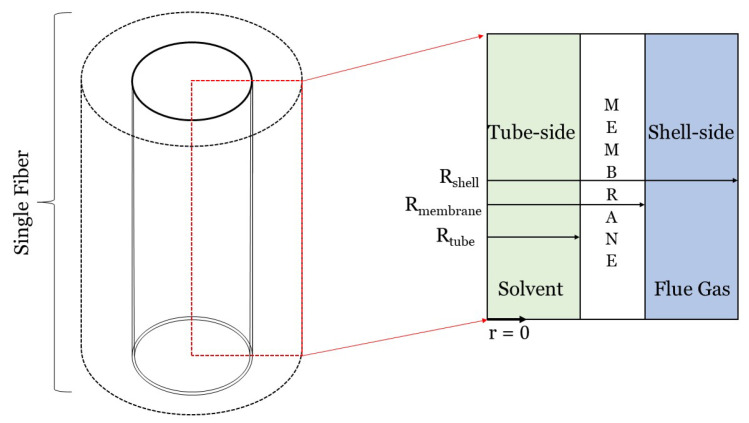
Graphic of the one-dimensional modeling framework for a gas-liquid HFMC. The radial dimension is the one dimension of interest; variations in the axial and angular dimensions are not taken into account. Liquid solvent flows through the inside or ”tube-side” of the fiber, while flue gas flows outside or on the ”shell-side” of the fiber.

Dimensionality reduction is a convenient, established, and powerful method for simplifying the system of the coupled partial differential equations (PDEs) that need to be solved for an adequate modeling representation of physico-chemical phenomena and devices, decreasing drastically the computational cost of any numerical implementation. If symmetry around an axis along the longitudinal direction of a HFMC can be justified, a 3D hollow fiber can be as reduced and effectively studied by a 2D model, where angular variations can be ignored for both concentration and velocity, e.g., Reference [23,31,60,61,62,63,64,65]. Further simplification can be achieved, if translational invariance for the fluid flow is also imposed upon our model along the longitudinal direction, making the axial component of the velocity dependent only on the radial coordinate [66]. An additional reasonable assumption can also be made based on the fact that the dominant component of the mass-transfer driving force, is usually perpendicular to the direction of the flow [55]. All the above taken together, result in an 1D model, where angular and axial variations of mass transfer can be neglected [67]. This section focuses on 1D models of carbon capture HFMCs. As shown in Figure 2, the radial dimension, or *r*, is typically the one dimension that is resolved in 1D HFMC models [16,18,26]. Although the flue gas and solvent streams can flow on either side of the fiber, we assume in Figure 2 and the following equations that solvent flows on the tube-side and that flue gas flows on the shell-side, like, for example, in Reference [60]. All theory presented in this paper will consist of three domains: the tube, membrane, and shell domains. One-dimensional models of HFMCs should either include a mass transfer coefficient or a model that describes the permeate(s) passing through the membrane. This section will focus first on using the resistance-in-series method to calculate mass transfer coefficients. The solution-diffusion and pore flow models will then be discussed briefly.

### 3.1. Resistance-In-Series (RIS) Model

**Figure 3:**
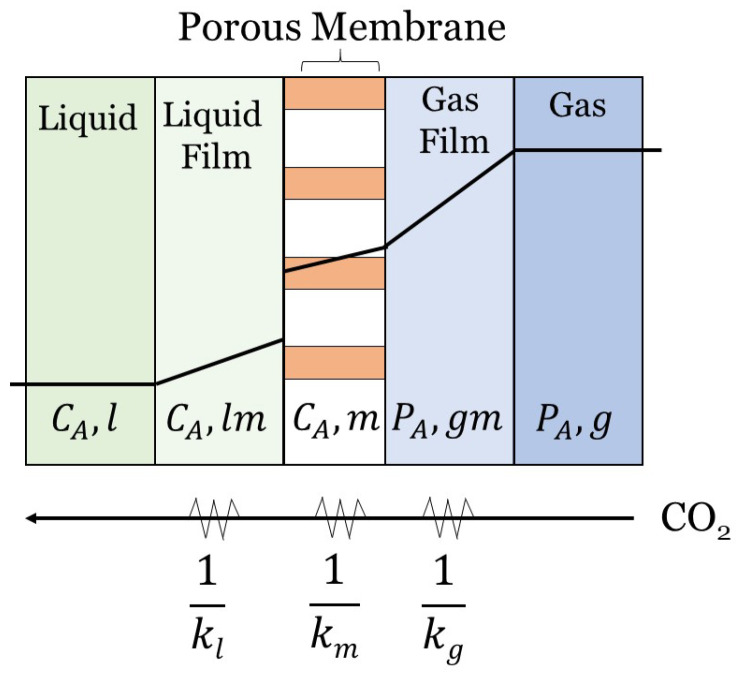
A resistance-in-series (RIS) illustration for CO${}_{2}$ crossing a membrane in a HFMC. There are mass transfer resistances associated with the gas phase, the membrane, and the liquid phase. Each resistance can be expressed as the inverse of the mass transfer coefficient for that phase. The layers included to accomplish this analysis are the gas, gas film, membrane, liquid film, and liquid.

The resistance-in-series (RIS) approach is often used to model the overall mass transfer coefficient (MTC) of individual fibers. For the purpose of this review paper, the RIS model will be considered as a 1D model with the mass transfer occurring in the radial direction. The RIS method is widely applicable to HFMC models and can even be used to model multi-stage HFMCs and a range of flow patterns [18,68]. It is a simple, effective means of determining the rate of CO${}_{2}$ transfer in HFMCs. The RIS method relies on mass transfer resistances, which are analogous to electrical resistors wired in series [69]. RIS is a simple method that breaks down the mass transfer process in a carbon capture HFMC into a series of steps: CO${}_{2}$ diffusion through the gas, CO${}_{2}$ diffusion through the membrane, and CO${}_{2}$ diffusion through the liquid (as shown in Figure 3). The RIS model for HFMCs is derived from double-film theory, where two fluids (i.e., two films) are considered with a membrane in between [7]. The double-film theory describes mass transfer resistance at the interface(s), or film(s), of the liquid, porous media, and gas phases [19]. Figure 3 illustrates the mass transfer through a non-wetted porous membrane for the three domains, where $P_{A}$ is the partial pressure of component A in the gas phase, and $C_{A}$ is the liquid concentration of component A.

The RIS model calculates the overall mass transfer coefficient (MTC) that is needed to calculate the flux:

(10) $$J_{CO_{2}}=k_{ov}\Delta C_{CO_{2}}.$$

The following assumptions and law’s are used to derive the overall MTC ($k_{ov}\left[\frac{m}{s}\right]$) [70]. A phase equilibrium is assumed at the gas-liquid interface. Fick’s first law of diffusion (Equation (3)) and Henry’s law (Equation (11)) for gas-liquid systems are also applied to derive the final flux equation:

(11) $$H=\frac{C_{CO_{2},l}}{P_{CO_{2},g}}.$$

Equation (3) is Fick’s first law of diffusion, where the flux is defined as the moles of a species moving past a region over a given length. Equation (11) shows the relationship between the concentration of CO${}_{2}$ and the partial pressure of CO${}_{2}$. This relationship produces Henry’s constant, *H*, to account for physical solubility of CO${}_{2}$ at the gas-membrane interface. Luis et al. [7] goes through the steps of applying these assumption’s and law’s to derive the overall MTC and the final flux equation (Equation (10)).

With each layer that $CO_{2}$ diffuses through in Figure 3, the mass transfer resistance of that layer is the inverse of the mass transfer coefficient of that layer $\left(R_{i}=1/k_{i}\right)$. Henry’s constant (*H*) must also be included in the domain interface resistance to account for solubility of gas in the liquid phase. The overall MTC can then be determined by summing these three resistances, as shown in Equation (12):

(12) $$\frac{1}{k_{ov}}=\frac{1}{k_{l}}+\frac{1}{k_{m}}+\frac{H}{k_{g}}.$$

Equation (12) expresses the overall MTC, $k_{ov}\left[\frac{m}{s}\right]$, as a function of the MTCs of the liquid, membrane, and gaseous phases: $k_{l}$, $k_{m}$, and $k_{g}$, respectively [71,72,73]. The membrane MTC, $k_{m}$, is determined by Equation (13):

(13) $$k_{m}=\frac{D_{eff}}{\delta },$$

where the effective diffusivity in the membrane, $D_{eff}=D_{CO_{2}}\frac{\varepsilon }{\tau }$, is defined as the ratio of the porosity to tortuosity of the membrane in $\left[\frac{m^{2}}{s}\right]$, and δ[m] is the thickness of the membrane [74]. $D_{eff}$ is determined using the Fickian, Maxwellian (if it is a multi-component system), and Knudsen gas diffusion coefficients [75]. The gas- and liquid-layer MTCs, $k_{g}$ and $k_{l}$, are determined using the following correlations:

(14) $$k_{g}=\frac{ShD_{CO_{2},g}}{d_{h}},$$

(15) $$k_{l}=\frac{ShD_{CO_{2},l}}{d_{i}},$$

where Sh is the Sherwood number, $d_{i}\left[m\right]$ is the inner fiber diameter, and $D_{CO_{2},g}$ and $D_{CO_{2},l}$ are the diffusivities of CO${}_{2}$ in the gas and liquid domains, respectively, in $\left[\frac{m^{2}}{s}\right]$. Equations (14) and (15) assume that liquid flows inside the fibers while gas flows on the shell side, as shown in Figure 2. If gas is flowing on the tube side and gas through the fibers, then the $d_{i}$ and $d_{h}$ terms in these equations need to be swapped. The gas and liquid MTCs should be modeled with respect to the system setup to consider the correct diameters ($d_{i}$ or $d_{h}$). There is no standard for where the solvent should flow (either the tube- or shell-side), but the hydraulic diameter should be modeled accordingly. Zhao et al. discuss the advantages and disadvantages of modeling the solvent on the tube- and shell-side [44].

The following correlations proposed by Yang and Cussler can be used to determine the Sherwood number for laminar flow on the tube-side and the shell-side of the fibers, respectively [76]:

(16) $$Sh_{tube}=1.62{\left(\frac{d_{i}Re}{L}\right)}^{0.33},$$

(17) $$Sh_{shell}=1.25{\left(\frac{d_{h}Re}{L}\right)}^{0.93}Sc_{CO_{2}}^{0.33},$$

where L[m] is the length of the fiber; $Sc_{CO_{2}}=\frac{\nu_{CO_{2}}}{D_{CO_{2}}}$ is the Schmidt number defined using the kinematic viscosity, $\nu [\frac{m^{2}}{s}]$; $Re=\frac{\rho Vd}{\mu }$ is the Reynolds number defined using the dynamic viscosity, ν[Pa·s]; $d_{h}$ is the hydraulic diameter; and $d_{i}$ is the inner diameter. These Sherwood correlations can be applied to gas or liquid phase flows on either side of the membrane. These correlations are specific to the geometric configuration shown in Figure 2, where solvent flows on the tube-side and flue gas flows on the shell-side of a cylindrical fiber. Additional mass transfer correlations for different configurations are covered in Table 2 of Cui et al.’s review paper on HFMCs for CO${}_{2}$ capture [77].

#### 3.1.1. Modeling Chemical Reactions in RIS

If a chemical reaction needs to be modeled, this can be incorporated into the RIS model by using a dimensionless enhancement factor, *E*. The enhancement factor is the ratio of mass transfer rate with reaction to the mass transfer rate without reaction. Equation (12) can thus be modified to incorporate the CO${}_{2}$ absorption reaction [78,79], as shown in Equation (18):

(18) $$\frac{1}{k_{ov}}=\frac{Hd_{o}}{k_{l}d_{i}E}+\frac{d_{o}}{k_{m}d_{lm}}+\frac{1}{k_{g}}.$$

The inner, outer, and mean logarithmic diameters of the fiber ($d_{i},d_{o},d_{lm}$) are also incorporated into this equation to account for the different diameters where each mass transfer step occurs [7]. For a system considering only a physical absorption, E=1 [80]. For a system considering chemical solvents, *E* is determined by the infinite enhancement factor ($E_{\infty }$) and the Hatta number (Ha) [81]. Zhao et al. [44], Cussler et al. [80], and Gaspar et al. [81] provide more information on how to incorporate the enhancement factor into the RIS equation when the reaction is the limiting, partially limiting, or not the limiting step in the overall mass transfer process.

Incorporating an enhancement factor into the RIS model is just one technique for modeling reaction chemistry in HFMCs. However, more often than not, the overall MTC determined by RIS is coupled with a separate solvent reaction chemistry model, as presented in Section 4. This approach is more accurate than using an enhancement factor because it incorporates a more detailed set of reactions coupled with transport and conservation equations. Details on how to model the absorption reaction on the liquid side for 1D models using common solvents can be found in Reference [29,82].

#### 3.1.2. Modeling Membrane Wetting in RIS

The examples and discussion so far have focused on ideal HFMCs. Actual HFMCs may experience membrane wetting over time, as reported in some HFMC experiments [26,83]. Membrane wetting is due to the surrounding environment, chemical reactions, changes in geometry (such as pore swelling), and capillary condensation [84]. To model membrane wetting in HFMCs, the following assumptions must be made: (1) membrane thickness, δ, is the total pore length, and (2) pores have a doughnut structure (in order to apply the Laplace-Young equation).

For 1D HFMC models, membrane wetting can be incorporated into the RIS equation. The RIS equation for a fully wetted membrane with reaction chemistry is given by [7]:

(19) $$\frac{1}{k_{ov}}=\frac{Hd_{o}}{k_{l}d_{i}E}+\frac{Hd_{o}}{k_{m}d_{lm}}+\frac{1}{k_{g}},$$

where $k_{m}$ is now defined using the thickness of the wetted part of the membrane, $\delta_{wetted}$ [85]:

(20) $$k_{m}=\frac{D_{eff}}{\delta_{wetted}}.$$

**Figure 4:**
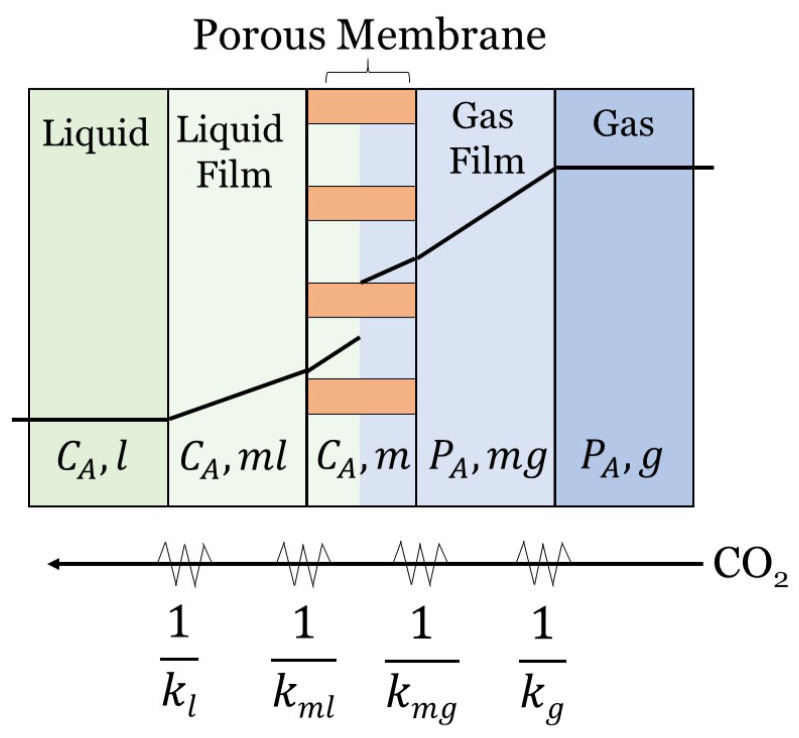
A resistance-in-series illustration for CO${}_{2}$ crossing a membrane in a HFMC with partial membrane wetting, where both liquid and gas fill the membrane pores.

However, a more realistic model is a partially wetted HFMC, where the pores are filled with both gas and liquid [86]. In this case, additional resistances (as shown in Figure 4) can be added to the equation using a parameter known as the wetting ratio:

(21) $$x^{*}=\frac{V_{w}}{V_{f}},$$

where $V_{w}$ is the volume of liquid in the pore, and $V_{f}$ is the pore volume of the membrane, given by $V_{f}\;=n_{p}\pi \varepsilon \left(r_{o}^{2}-r_{i}^{2}\right)L$. The pore volume of the membrane depends on the number of pores, $n_{p}$; the membrane porosity, ε; the fiber length, *L*; and the outer and inner radii of the fiber: $r_{o}$ and $r_{i}$, respectively.

The RIS equation for a partially-wetted membrane with reaction chemistry is given by:

(22) $$\frac{1}{k_{ov}}=\frac{Hd_{o}}{k_{l}d_{i}E}+\frac{Hd_{o}x^{*}}{k_{ml}d_{lm}}+\frac{d_{o}}{k_{mg}d_{lm}}\left(1-x^{*}\right)+\frac{1}{k_{g}},$$

where the term with $x^{*}$ accounts for the wetted portion of the membrane, and the term with $\left(1-x^{*}\right)$ accounts for the non-wetted portion of the membrane. $k_{ml}$ is the membrane mass transfer coefficient for the liquid-filled pores, and $k_{mg}$ is membrane mass transfer coefficient for the gas-filled pores. Sometimes these extra resistances can make the total membrane resistance account for >90% of the overall mass transfer resistance, which underscores the importance of accounting for membrane wetting in models [21,51]. Membrane wetting can be reduced or eliminated by using a dense skin layer [87]. This can decrease the overall MTC by two or three orders of magnitude [88].

When modeling membrane wetting effects, membrane parameters, such as pore size and distribution, must also be accounted for [16,17,72]. Membrane wetting can vary along the length of a membrane as transmembrane pressure varies along a membrane [44]. The transmembrane pressure is defined as the pressure difference across the membrane. Membrane wetting is determined by comparing the breakthrough pressure to the transmembrane pressure [89]. The breakthrough pressure, defined in Equation (23), should be used as the critical pressure to determine if the liquid will penetrate into the pores of the membrane and cause membrane wetting [7,90]:

(23) $$\Delta P=-\frac{4B\gamma cos\theta }{d_{max}}.$$

Equation (23), known as the Laplace-Young equation, depends on the surface tension of the liquid, $\gamma [\frac{N}{m}]$; the contact angle, θ; and the maximum pore diameter, $d_{max}\left[\mu m\right]$. $d_{max}$ is the critical pore diameter, which dictates whether or not membrane pores are wetted. If membrane pore diameter is greater than or equal to $d_{max}$, then the pores should be modeled as wetted [16,17]. Pore shape is accounted for in the pore geometry coefficient, *B*, where B=1 for perfectly cylindrical pores, and 0<B<1 for non-cylindrical pores [44].

### 3.2. Solution-Diffusion Model

**Figure 5:**
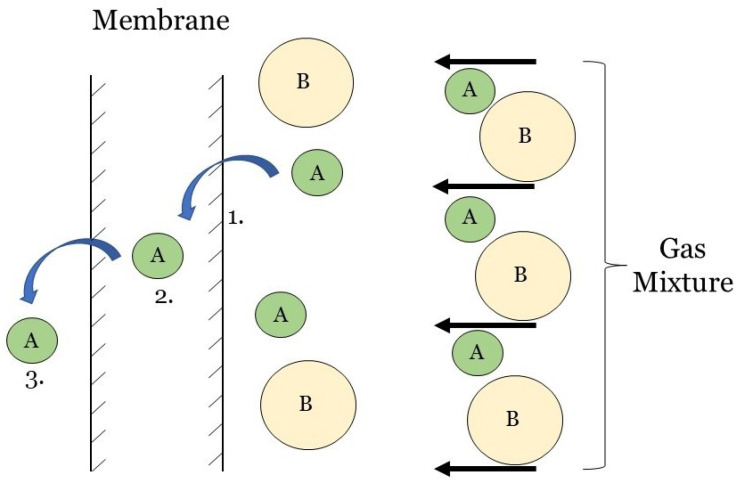
Graphical depiction of the solution-diffusion three-step process in a carbon capture HFMC for gas mixture of molecule A and B: 1. Molecule A sorption at the gas-membrane interface, 2. Molecule A diffusion through the membrane, and 3. Molecule A desorption at the solvent-membrane interface. The permeants are separated because of the differences in the solubilities and the variations in the diffusive rates of the different flue gas species in the membrane [92,93].

The solution-diffusion model is a standard 1D model for dense, non-porous polymeric membranes and as such is not frequently encountered for modeling HFMCs. The permeants are separated because of the differences in the solubilities and the variations in the diffusive rates of the different flue gas species in the membrane. This transport mechanism occurs in the reverse osmosis, pervaporation, and polymeric gas separation membranes. The lack of micro-pores, reasonably justifies the assumption of constant pressure throughout the membrane and the concentration difference being the mass-transfer’s driving force [91]. This model follows gaseous CO${}_{2}$ as it undergoes a three-step process (shown in Figure 5): (1) CO${}_{2}$ sorption onto the membrane’s on the gas side, (2) CO${}_{2}$ diffusion through the membrane, and (3) CO${}_{2}$ desorption from the solvent side of the membrane [92,93].

To apply the solution-diffusion model, the following assumptions must be made: (1) the rates of absorption and desorption at the interfaces are much higher than the rate of diffusion through the membrane, (2) equilibrium is assumed between fluids and membrane at the interfaces, (3) there are no visible pores, (4) pressure within the membrane is constant, and (5) the chemical potential is represented only by the concentration gradient. The solution-diffusion model is naturally characterized by Fick’s first law of diffusion (Equation (3)), which gives the flux of a gaseous species as a function of concentration gradient [94]. Notwithstanding the fact that solution-diffusion model is not endemic to HFMC, it has been reported to predict HFMC behavior in industrial settings [95] and in power plants [91]. The solution-diffusion model is also a quick way to represent membranes to perform membrane system optimization [96] and minimize failure [97,98]. It has also been used to model multi-component flue gas systems and non-isothermal conditions [99].

### 3.3. Pore Flow Model

**Figure 6:**
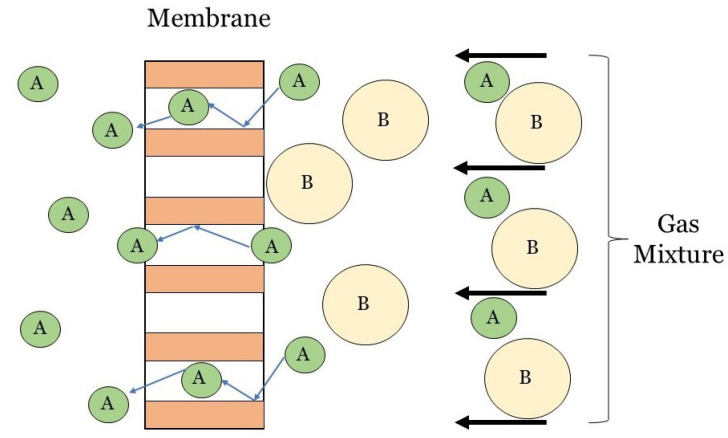
Graphical depiction of the pore flow model, where molecule A crosses the membrane due to a pressure difference. The illustration is not drawn to scale to emphasize the flow through permanent pores [70,94].

Another 1D membrane modeling approach is the pore flow model. Unlike dense polymeric membranes, for applications, like ultrafiltration, microfiltration, and microporous gas flow membranes, the membrane consists of a network of micro-pores. The separation is achieved as a result of filtering, as only species with molecular sizes less than the size of the pores can permeate through the membrane. A direct consequence of the separation mechanism is that the concentration in the membrane is assumed constant, and gas transfers across the membrane by pressure-driven convective flow through the pores as shown in Figure 6 [70,94]. The pore-flow model can be described on the macro-scale by Darcy’s law. For $CO_{2}$ as the permeating species:

(24) $$J_{CO_{2}}=-\frac{k_{D}}{\mu }C_{CO_{2}}\frac{\partial P}{\partial x},$$

where $k_{D}\left[m^{2}\right]$ is the Darcy’s Law coefficient, $\frac{\partial P}{\partial x}\left[\frac{Pa}{m}\right]$ is the pressure gradient across the membrane thickness, μ[Pa·s] is the viscosity, and $C_{CO_{2}}\left[\frac{mol}{m^{3}}\right]$ is the concentration. Both the solution-diffusion and pore flow model are capable of modeling 1D gas transport through a membrane. The main distinction between the two is that concentration difference drives transport in the solution-diffusion model, whereas pressure difference drives transport in the pore flow model. Additionally, the membrane material (i.e., the size and permeance of the pores) is an important factor to consider as it determines which model should be used. The solution-diffusion model is recommended for membranes with pore sizes below 5 Å, while the pore flow model is recommended for membranes with pore sizes of 10–1000 Å.

The RIS method, solution-diffusion model and pore flow model can each accurately represent the physics of CO${}_{2}$ removal from flue gas in HFMCs. The RIS method uses an estimated overall MTC to estimate the CO${}_{2}$ flux across the membrane. The solution-diffusion and pore flow models, on the other hand, do not require a MTC to be calculated. Instead, the solution-diffusion model relies on Fick’s first law of diffusion to represent the permeance of CO${}_{2}$ through the membrane as a function of concentration, and the pore flow model uses Darcy’s law and pressure difference to determine CO${}_{2}$ flux. RIS is the most popular method for 1D modeling of HFMCs. The RIS method has the advantage of being able to represent membranes that have both porous and nonporous portions [100]. One-dimensional models using these three approaches can accurately match experimental results, as well as 2D and 3D models [101,102]. The main drawback of 1D models is that they cannot capture more complex dynamics inside a HFMC fiber or bundle (e.g., concentration profiles, flow distributions), which is where 2D and 3D models add value.

## 4. Two-Dimensional Modeling

**Figure 7:**
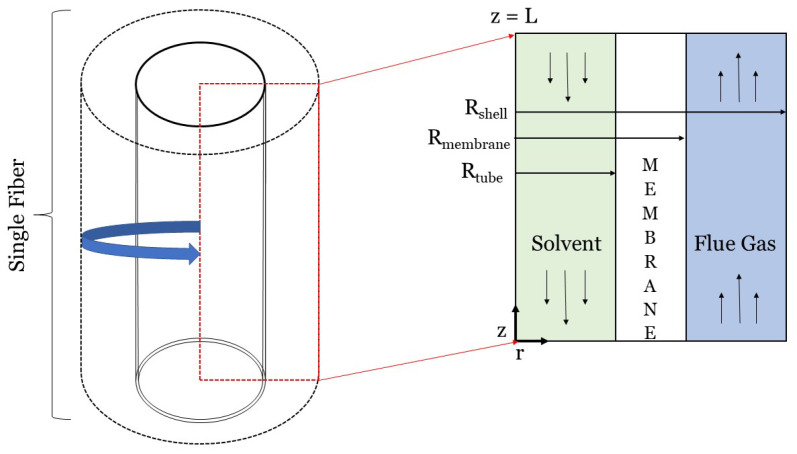
Graphic of the two-dimensional axisymmetrical modeling framework for a gas-liquid HFMC. The axial and radial dimensions are the dimensions of interest; variations in the angular dimension are not considered due to symmetry. In this graphic, solvent flows on the tube-side, while flue gas flows counter-flow on the shell-side.

Two-dimensional modeling of carbon capture HFMCs typically consists of modeling a single hollow fiber. However, 2D modeling can more precisely account for physics and transport than 1D models by incorporating axial diffusion and convection [28,32,103]. To computationally model axial and radial mass transfer characteristics (diffusion, convection, and chemical reactions), a complete mass transfer analysis is necessary using governing equations and constitutive laws. Figure 7 will be used throughout this section to walk through the theory that should be followed to complete a mass transfer analysis for a HFMC. Figure 7 illustrates the 2D modeling framework for a single hollow fiber in a counter-flow HFMC, where liquid solvent flows on the tube-side and flue gas flows on the shell-side. Once the general governing equations and laws are introduced, the equations and laws will be applied to the system presented in Figure 7. These equations will then be modified to account for membrane wetting. It should be noted that this section will demonstrate computation fluid dynamics (CFD) modeling theory behind a single 2D-axisymmetric hollow fiber. If one desires to develop a 2D model of a HFMC bundle, the results can simply be multiplied by the number of fibers in the bundle.

### 4.1. Governing Equations for a 2D-Axisymmetric HFMC Fiber

Using as starting point the balances of mass, momentum and energy presented in Section 2 accompanied by the relevant constitutive laws and applying a set of assumptions, one can get to a system of partial differential equations that mathematically represent the physical setup. For the purposes of this analysis a popular set of assumptions comprises: steady state operation, rotational symmetry for both velocity and concentration fields, translational invariance for the fluid flow, constant material properties leading to incompressibility in the form of ∇u=0, and constant membrane properties. The following equations are based on the cylindrical coordinates framework presented in Figure 7. The general mass balance for this system is given by Equation (25) and is derived from Equation (6) for a 2D-axisymmetric hollow fiber [55].

(25) $$v_{z}\frac{\partial C_{A}}{\partial z}-r_{A}=D_{A}\frac{\partial^{2}C_{A}}{\partial r^{2}}+\frac{D_{A}}{r}\frac{\partial C_{A}}{\partial r}+D_{A}\frac{\partial^{2}C_{A}}{\partial z^{2}},$$

where $D_{A}\left[\frac{m^{2}}{s}\right]$ and $C_{A}\left[\frac{mol}{m^{3}}\right]$ are the diffusion coefficient and the concentration of species A, respectively; $v_{z}\left[\frac{m}{s}\right]$ is the axial velocity component; and $r_{A}\left[\frac{mol}{m^{3}s}\right]$ is the reaction rate of species A. Equation (25) will now be applied to each domain of the system (e.g., tube, membrane, shell) and modified to represent the physics occurring within each domain.

Modeling diffusion in the membrane domain is the simplest application because the chemical reaction rate and convection terms drop out. Therefore, Equation( 25) reduces to Equation (26) within the membrane:

(26) $$0=D_{CO_{2}-mem}\left(\frac{\partial^{2}C_{CO_{2}}}{\partial r^{2}}+\frac{1}{r}\frac{\partial C_{CO_{2}}}{\partial r}+\frac{\partial^{2}C_{CO_{2}}}{\partial z^{2}}\right).$$

In comparison, when Equation (25) is applied to the tube- and shell-side, additional mass transfer terms must be included. The 2D mass transport equations for the tube- and shell-side, respectively, are shown below [62,64,104,105]:

(27) $$v_{z,tube}\frac{\partial C_{CO_{2}}}{\partial z}-r_{CO_{2}}=D_{CO_{2}-tube}\left(\frac{\partial^{2}C_{CO_{2}}}{\partial r^{2}}+\frac{1}{r}\frac{\partial C_{CO_{2}}}{\partial r}+\frac{\partial^{2}C_{CO_{2}}}{\partial z^{2}}\right),$$

(28) $$v_{z,shell}\frac{\partial C_{CO_{2}}}{\partial z}=D_{CO_{2}-shell}\left(\frac{\partial^{2}C_{CO_{2}}}{\partial r^{2}}+\frac{1}{r}\frac{\partial C_{CO_{2}}}{\partial r}+\frac{\partial^{2}C_{CO_{2}}}{\partial z^{2}}\right).$$

The reaction in the system only occurs within the solvent. Therefore, the reaction rate term $\left(r_{CO_{2}}\right)$ is omitted from the shell-side equation because that is the gas domain. However, $r_{CO_{2}}$ is included in the tube-side equation to represent the rate of CO${}_{2}$ absorption into the solvent. Although reaction kinetic models for determining $r_{CO_{2}}$ are beyond the scope of this paper, it is important to note that $r_{A}$ is a potential source of non-linearity. For example, if the solvent has a reaction constant that is second or third order, the equation will become nonlinear. This does not only mean that analytical solutions are impossible to get. Even more, non-linearity results in systems of stiff equations, challenging the most advanced, state-of-the-art solvers and numerical algorithms, often rendering the problems untraceable. Other research groups have incorporated chemical absorption into their 2D HFMC models introducing Monoethanolamine (MEA), Methyldiethanolamine (MDEA), and Diethanolamine (DEA), respectively [27,32,106].

The fluid velocities on the shell and tube sides, $v_{z,shell}$ and $v_{z,tube}$, are needed to solve Equations (27) and (28). These velocities are determined by using the 2D cylindrical Navier–Stokes and continuity equations based on Equations (7) and (8) [30,62].

(29) $$\rho \left(v_{r}\frac{\partial v_{r}}{\partial r}+v_{z}\frac{\partial v_{r}}{\partial z}\right)=-\frac{\partial P}{\partial r}+\mu \left(\frac{1}{r}\frac{\partial }{\partial r}\left(r\frac{\partial v_{r}}{\partial r}\right)-\frac{v_{r}}{r^{2}}+\frac{1}{r^{2}}\frac{\partial^{2}v_{r}}{\partial z^{2}}\right),$$

(30) $$\rho \left(v_{r}\frac{\partial v_{z}}{\partial r}+v_{z}\frac{\partial v_{z}}{\partial z}\right)=-\frac{\partial P}{\partial z}+\mu \left(\frac{1}{r}\frac{\partial }{\partial r}\left(r\frac{\partial v_{z}}{\partial r}\right)+\frac{\partial^{2}v_{z}}{\partial z^{2}}\right),$$

(31) $$0=\frac{1}{r}\frac{\partial (rv_{r})}{\partial r}+\frac{\partial v_{z}}{\partial z},$$

where $v_{r}\left[\frac{m}{s}\right]$ is the radial velocity vector, $v_{z}\left[\frac{m}{s}\right]$ is the axial velocity vector, μ[Pa·s] is the viscosity of the fluid, P[Pa] is the fluid pressure, and $\rho [\frac{kg}{m^{3}}]$ is the fluid density. These equations describe steady-state, laminar, incompressible flow of Newtonian fluids. The above equations will be further reduced as translational invariance and no body forces in the r-z plane, result in the $v_{r}$ component of the velocity to be zero and the only velocity component that remains is $v_{z}\left(r\right)$.

Analytically solving for the velocity profile on the tube-side, the Navier–Stokes and continuity equations result in the well-established Hagen-Poiseulle velocity profile [107]:

(32) $$v_{z,tube}\left(r\right)=2v_{avg,tube}\left(1-{\left(\frac{r}{R_{tube}}\right)}^{2}\right),$$

where $v_{avg,tube}\left[\frac{m}{s}\right]$ is the average velocity in the tube-side. The shell-side velocity profile is not as simple as the tube-side solution because it first requires a bundle approximation to determine the shell-side radius. In order to solve for the shell-side velocity profile, the following assumptions are made: (1) the fibers are evenly distributed in the shell space, (2) the bundle’s porosity is equal to the fluid’s envelope porosity, and (3) there is no friction on the shell side. Equation (33) defines the shell-side radius, $R_{shell}$, as a function of the volume fraction, φ:

(33) $$R_{shell}=R_{membrane}\sqrt{\frac{1}{1-\varphi }},$$

where the packing density of the membrane, φ, depends on the number of fibers in the bundle, *n*, and the radius of the membrane module, $R_{module}$. $R_{module}$ is defined as shown in Equation (34).

(34) $$1-\varphi =\frac{nr^{2}}{R_{module}^{2}}.$$

This relationship describes the volume fraction of the void. Once the shell-side radius is calculated according to Equation (33), the shell-side velocity profile is determined using Happel’s free surface model [108]. Happel’s free surface model describes the axial velocity for flow in the annulus between concentric cylinders as a function of the radial coordinate:

(35) $$v_{z}\left(r\right)=-\frac{1}{4}\mu \frac{\partial p}{\partial x}\left[\left(R_{tube}^{2}-r^{2}\right)+2R_{shell}^{2}ln\left(\frac{r}{R_{tube}}\right)\right].$$

Equation (35) is integrated with respect to *r* to give the volumetric flow-rate. The average velocity for a fixed pressure gradient is then obtained, by dividing by the cross-sectional area of the annulus, $\pi (R_{shell}^{2}-R_{tube}^{2})$:

(36) $$v_{avg}=-\frac{1}{8(R_{shell}^{2}-R_{tube}^{2})}\mu \frac{\partial p}{\partial x}\left[4R_{tube}^{2}R_{shell}^{2}-3R_{shell}^{4}-R_{tube}^{4}+4R_{shell}^{4}ln\frac{R_{shell}}{R_{tube}}\right].$$

Combining Equations (35) and (36), the analytical solution to Happel’s free surface model is given by:

(37) $$v_{z,shell}\left(r\right)=2v_{avg}f\left(r\right),$$

where f(r) is:

(38) $$f\left(r\right)=\left[1-{\left(\frac{R_{shell}}{R_{tube}}\right)}^{2}\right]\left[\frac{{\left(\frac{r}{R_{tube}}\right)}^{2}-{\left(\frac{R_{shell}}{R_{tube}}\right)}^{2}-2ln\left(\frac{R_{shell}}{r}\right)}{3+{\left(\frac{R_{shell}}{R_{tube}}\right)}^{4}-4{\left(\frac{R_{shell}}{R_{tube}}\right)}^{2}-4ln\left(\frac{R_{shell}}{R_{tube}}\right)}\right].$$

Equations (25)–(38) describe the flow and concentration distribution for a HFMC in the radial and axial dimensions.

Common concentration boundary conditions used in 2D axisymmetric hollow fiber models are shown in Table 3, where the inlet of the flue gas is at z=0 and the inlet of the solvent is at z=L. The physical solubility of CO${}_{2}$ in the solution is defined as $m[\frac{mol}{L}]$. These boundary conditions are applied to the governing equations in the previous subsection to solve the equations analytically or numerically. For example, Equation (25) can be coupled with boundary conditions from Table 3 to solve for CO${}_{2}$ transport.

In addition to the transport and conservation equations, 2D HFMC models should account for reaction chemistry on the permeate side. Many 2D models use water as their initial solvent to study diffusion and convection effects without the confounding effect of chemical reactions [36,37]. Building on those results, the chemical reactions are then incorporated into models to study the chemical adsorption of common solvents, such as MDEA [30,31,109], DEA [27], and MEA [20,22]. For example, Shirazian et al. used this 2D modeling approach to compare different solvents and found that MEA is a better solvent than MDEA based on CO${}_{2}$ absorption [110]. Other modeling studies compare common solvents [23,33], blended solvents [24,111,112], and ionic liquids [113] for optimized CO${}_{2}$ separation. These reaction models can be found in the previously cited papers and incorporated into the reaction rate constant in Equation (27).

Many 2D models carbon capture HFMC models have been used to study the impact of operating conditions on separation performance. This includes varying solvent and gas flow-rates to study how much raising the solvent:gas flow-ratio boosts CO${}_{2}$ removal rates [14,43]. Other operating conditions like pressure [15], solvent composition [114], flow direction [35], and membrane permeability [60] have been varied to study their effect on carbon capture HFMC performance using the 2D mass balance approach. Some groups have also developed nonisothermal 2D HFMC models to study how the heat released by CO${}_{2}$ absorption impacts performance. Temperature variation within a HFMC can lead to evaporation and condensation within the pores, which impacts membrane performance. Nonisothermal 2D HFMC models must incorporate the thermal energy equation (Equation (9)) to account for heat release and temperature variations [44,111]. Another approach is to consider the temperature effects only on the solvent-side of the HFMC, where the CO${}_{2}$ binds to the solvent and needs to undergo stripping. In this case, the temperature effect can be added in the reaction rate expression from Equation (27) as a function of temperature and concentration.

### 4.2. 2D Modeling of Membrane Wetting

As mentioned in Section 3, if the transmembrane pressure surpasses the breakthrough pressure, wetting must be considered in the model. For 2D models, wetting effects can be applied to the governing 2D equations presented above. For a partially wetted system, two additional equations are introduced to account for the diffusion within a pore that is partially filled with gas and the solvent [63]. For the gas filled pore, the membrane mass transfer equation considered only diffusion.

(39) $$0=D_{CO_{2},mg}\left(\frac{\partial^{2}C_{CO_{2},mg}}{\partial r^{2}}+\frac{1}{r}\frac{\partial C_{CO_{2},mg}}{\partial r}+\frac{\partial^{2}C_{CO_{2},mg}}{\partial z^{2}}\right).$$

For the liquid filled pore, the mass transfer equation consists of diffusion and the reaction between CO${}_{2}$ gas molecules and the solvent.

(40) $$0=D_{CO_{2},ml}\left(\frac{\partial^{2}C_{CO_{2},ml}}{\partial r^{2}}+\frac{1}{r}\frac{\partial C_{CO_{2},ml}}{\partial r}+\frac{\partial^{2}C_{CO_{2},ml}}{\partial z^{2}}\right)+r_{CO_{2},ml}.$$

It should be noted that, with two additional equations, Equations (39) and (23), four more boundary conditions need to be included in the gas-membrane and liquid-membrane interfaces, as listed in Table 4 [109].

For more information on modeling membrane wetting, refer to the following review papers [44,86,89,115]. More often than not, membrane wetting is assumed to be negligible. However, several models do incorporate membrane wetting [22,25,63,116,117]. Membrane wetting is an important phenomenon that should be accounted for in more carbon capture HFMCs, particularly in situations where membrane wetting is known to occur experimentally.

### 4.3. Benefits of 2D Axisymmetric Modeling

**Figure 8:**
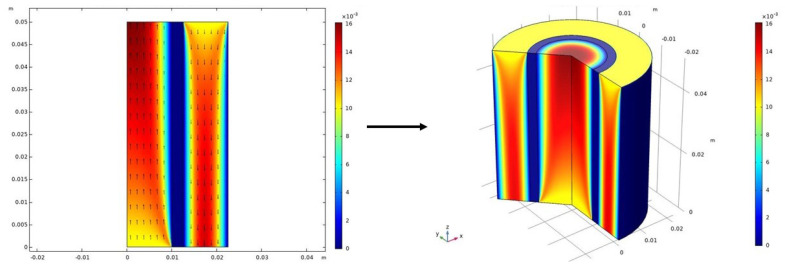
Velocity profiles in a counter-flow, gas-liquid HFMC. The liquid flows on the tube-side, entering at z=L, and the gas enters the shell-side at z=0. This 2D-axisymmetric model resolves properties in a 2D cross-section (**left**), then revolves those results around the z-axis to form a 3D plot (**right**). These images were produced using COMSOL Multiphysics 5.5.${}^{®}$).

Two-dimensional axisymmetric HFMC models have led to greater understanding of mass transport phenomena (e.g., convection, diffusion, chemical effects) within an individual fiber. These models can produce 3D visualizations by revolving the 2D results around the z-axis, as shown in Figure 8. This 2D axisymmetric approach is sufficient for most cases, where angular variations are negligible. Two-dimensional axisymmetric modeling offers many of the benefits of a 3D model (e.g., visualizing concentration variations throughout the fiber) without the added computational burden of running a full 3D model. This 2D-axisymmetric approach has also been applied to model concentration distribution within a HFMC bundle using the mass balance equations presented earlier [118]. A full 3D model that resolves the physics in the angular direction, as well, can, in principle, be more accurate than the 2D-axisymmetric approach, if there is substantial evidence that the symmetry assumption breaks down. This, for example, could be the case if turbulent flows are induced, since turbulent eddies and the dissipation of energy are inherently 3D structures and effect correspondingly. However, many researchers infer that the angular variation is not critical to the results at hand and choose the 1D or 2D-axisymmetric routes instead.

Although modeling in 2D requires more complex equations and computational power than 1D modeling, the results are more accurate and can better reproduce experimental data for a single fiber. Two-dimensional-axisymmetric models of HFMCs can also be used to study and predict specific phenomena that cannot be explored in 1D, such as slow kinetic reactions and axial convection and diffusion effects. In general, 2D models can offer information for a single fiber, but their results are not immediately transferable to HFMC bundles. However, 3D modeling is often necessary to obtain higher accuracy solutions and resolve more detailed 3D phenomena that cannot be captured with 2D models, e.g., turbulence, flow maldistributions, non-uniform material properties, etc.

## 5. Three-Dimensional Modeling

**Figure 9:**
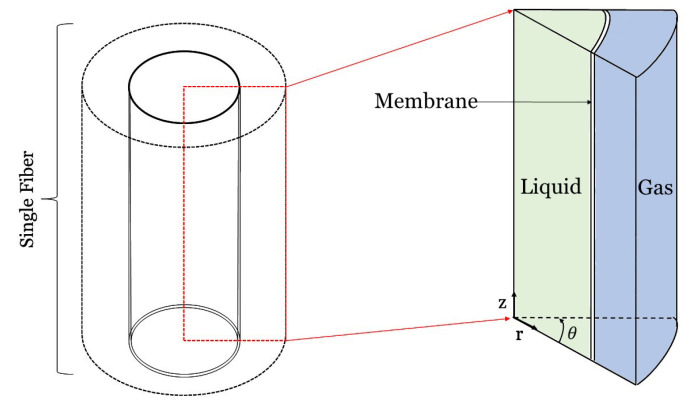
Graphic of the three-dimensional modeling framework for a gas-liquid HFMC. Axial (z), radial (r), and tangential (θ) variations are all resolved. In this graphic, solvent flows on the tube-side while flue gas flows on the shell-side.

Three-dimensional modeling of HFMCs is the best way to describe 3D phenomena, ensure the highest degree of accuracy, and capture angular variations along with axial and radial variations. Three-dimensional models are mostly useful when studying non-uniformity throughout a bundle (e.g., non-uniform flow distribution near ports and in the case symmetry does not hold). Figure 9 shows these three dimensions (axial, radial, tangential) for a single hollow fiber. This section discusses 3D modeling of HFMCs at a high level because the governing equations and set-up for modeling a 3D HFMC fiber are similar to those presented in the 2D section but with additional angular equations and terms. The governing equations for 3D models are also often built into software packages, so they do not need to be coded from scratch. Multiphysics software packages (e.g., ANSYS, OpenFOAM${}^{®}$, and COMSOL Multiphysics ${}^{®}$) are often preferred to model these types of systems.

One of the main advantages of 3D modeling for HFMCs is that it can be used to study transport variations throughout an entire bundle, not just in a single fiber. Whereas 1D and 2D HFMC models are limited to a single fiber, 3D models can be developed for an entire HFMC bundle. These 3D bundle models can provide information about flow distribution throughout the bundle and be used to study the impacts of fiber non-uniformity. One team used this bundle approach in COMSOL Muliphysics${}^{®}$ to calculate the overall MTC to describe the fluid flow for an incompressible fluid [119]. Another research group investigated the effects of temperature variation on chemical reactions in a 3D HFMC bundle by directly coding the 3D energy conservation equations and RIS for heat transfer resistances [120]. There have also been studies that focus on radial variations in a bundle exposed to steady-state turbulent flow to determine the optimal fiber arrangement [121]. Although 3D bundle modeling is the most accurate and detailed approach, it is also the most computationally expensive approach mentioned so far.

One way to simplify the 3D HFMC bundle model (and reduce computational burden) is to treat the bundle as a homogeneous, porous media [122]. Darcy’s Law (Equation (24)), derived from a simplified Navier–Stokes equation, can be applied to the system to describe the pressure drop and fluid flow within a porous medium. This approach is extensively researched for packed bed models [123,124,125,126], and the same principles apply to flow through a bundle of fibers. The mass balances and MTCs in these porous media models are calculated using the approaches discussed in Section 3 and Section 4. This approach has also been successfully used in other research fields to model reverse-osmosis for water filtration [127,128] and blood oxygenation devices [129,130]. These models would provide more insight into the flow and concentration distributions within HFMC bundles, so future research is recommended in this area.

Three-dimensional models of HFMCs have improved significantly in the past decade due to advances in computational capabilities. However, there is still significant room for improvement in reducing computational cost of these models while maintaining accuracy. Although many 1D and 2D HFMC models have been developed for PCC, 3D HFMC models are relatively new and therefore require further development. The next section compares the 1D, 2D, and 3D modeling approaches for HFMCs in PCC with an emphasis on the assumptions made by each of these models.

## 6. Comparison of 1D, 2D, and 3D Modeling Approaches

Each of the three modeling approaches described in the previous sections has unique merits and drawbacks. This section will compare these three modeling approaches with an emphasis on the different assumptions made by each type. Table 5 compares state-of-the-art 1D, 2D, and 3D carbon capture HFMC models based on their controlling assumptions, which are listed below the table. Table 5 provides a foundation for researchers seeking to develop the best HFMC carbon capture model to meet their particular needs.

One-dimensional models are assumed to have perfect flow distribution, fiber alignment, uniform membrane properties, etc., 1D models assume that reactions and mass transfer occur within a very thin reaction "film," which effectively reduces the axial dimension and leaves only variation in the radial direction [131]. One-dimensional modeling is considered sufficient for many applications, particularly those that involve scaling-up to larger system sizes. One-dimensional modeling is especially well-suited for larger systems because it is computationally expensive to resolve 2D or 3D effects in large HFMC systems. Many researchers prefer 1D models to 2D models for the PCC HFMC application, because 1D models are significantly faster and provide sufficiently accurate results [131]. Overall, 1D models can predict the results from previous experiments and simulations in 2D and 3D [101]. However, 1D modeling is only applicable if the MTCs are known or defined experimentally. If the MTCs are not known prior to the analysis, 2D or 3D modeling techniques may be necessary to determine the overall MTC because they are easier and cheaper than experimentation. Obtaining MTCs from experimental data is not an easy task, since one needs to accurately track the interfacial area between liquid and gas. This requirement in its turn demands state-of-the-art imaging techniques, without which the only way to predict MTCs, necessary for any scale-up attempt or actual system-design effort, is futile. Two-dimensional-axisymmetric and 3D models of a single fiber can give access to the fiber’s MTC, while 3D models of the whole bundle can generate the bundle’s average MTC.

Most HFMC modeling work thus far has focused on 1D because it is the simplest approach and provides sufficient accuracy for many applications. For example, one comparison between 1D and 3D HFMC models found only a 2 percent deviation in mass balance results [102]. This suggests that unless the researcher is looking for more detailed information internal to the fiber or bundle, a 1D model should be sufficiently accurate. Another downside of 1D models, besides the fact that it requires pre-determined MTC, is that it is unable to resolve 2D or 3D effects, such as fluid swirling or CO${}_{2}$ concentration gradients. It is possible to overcome some 1D limitations by enhancing 1D models (e.g., by adding wetting effects). However, some membrane systems are best modeled in 2D or 3D to capture radial and/or angular effects. Table 5 provides common assumptions used for 1D HFMC models in reference to the list (listed after Table 5) in the beginning of this section. The prevalent assumptions among 1D models are assumptions 3–6 and 8–9, where the physics focuses on the membrane interface.

As seen in Table 5, assumptions 10–15 are common assumptions made within 2D models. For example, most 2D species calculations rely on Happel’s free surface model offering an analytical calculation of the shell-side velocity profile, as presented in Section 4. Other assumptions include zero inlet CO${}_{2}$ concentration on the solvent side. These assumptions allow the modeler to successfully solve the fundamental laws and governing equations, simplifying the simulation of a HFMC. Zaidiza et. al. provide a more thorough comparison of the 1D and 2D modeling approaches for PCC HFMCs [97]. Another approach that combines the advantages of the two modeling approaches is hybrid 1D-2D modeling. For example, Chabanon et al. [19] uses a 1D model for the gas-side and a 2D model for the liquid-side. There is more work in the literature that covers hybrid 1D-3D modeling, such as Bao et al. [132], that studies the correlations of mass transfer coefficients between regularly and randomly packed bundles for gas-gas HFMCs.

Table 5 also provides common assumptions used for 3D models, including Happel’s free surface model and zero initial concentration on the solvent side. Although 3D models of HFMCs are less common than 1D and 2D models, they are growing in popularity as advanced algorithms, hardware and software are facilitating 3D modeling simulations. Three-dimensional models of HFMCs bundles, enable researchers to go beyond modeling a single hollow fiber and explore non-uniform effects. Three-dimensional bundle models are recommended for modelers looking to transfer their work into a process or system level simulation for this important technology of CC, assuming they can be computationally handled. Challenges however still remain due to the numerical cost of the calculations [133].

Modeling the bundle as a porous medium, though not common in the carbon capture field, there are other of scientific applications we could get inspiration from. Mazaheri et al. [134] offer a comparison between fiber modeling and porous media for a blood oxygenator device, finding that the velocity distributions are different and the porous media approach may lead to errors when calculating the transport properties. Three-dimensional bundle models can provide useful information about end effects and the impact of manifolds on flow distribution. However, modelers must prioritize the outcome of the project and decide if simplifying a 3D bundle using the porous media approach is right for them, or if they should simplify their model to 1D or 2D. Overall, 3D models are powerful tools for determining how conditions vary throughout a 3D bundle, not just along the length of an average fiber.

List of Assumptions in Table 5:

1. Steady-state, laminar, Newtonian, incompressible fluid, and plug flow with fully-developed velocity profiles.
2. Ideal gas law (assumes the gas particles are (1) in continuous, rapid motion, (2) are so small that their volume is negligible, (3) do not interact, and (4) temperature is proportional to the average kinetic energy); and Henry’s law (assumes constant temperature and that the vapor phase behaves as an ideal gas).
3. Fick’s law of diffusion (assumes constant diffusion coefficient) and thermal conductance through membrane, with adiabatic behavior.
4. Rate-controlled reversible reaction.
5. Heat and mass transfer are equal at the interface (condensation from the temperature difference occurs at the liquid-membrane interface).
6. Uniform membrane properties (constant tortuosity and distribution of membrane pore size, wall thickness, non-wetting).
7. Mass transfer between gas-liquid phases is a result of film diffusion.
8. Curvature effect of the membrane surface on mass transfer is negligible.
9. Happel’s free surface model (assumes the bundle’s porosity is equal to the fluid’s envelope porosity and assumes no friction on the shell-side).
10. Initial zero CO${}_{2}$ concentration on the solvent side.
11. Zero mass transfer at the two fiber ends.
12. Constant volumetric flow rate.
13. Large mass transfer rate between gas and liquid.
14. Non-wetted operation in which the gas mixture fills the membrane pores.
15. Ideal feed gas (fouling/pollution not accounted for).
16. Fibers are rigid walls (no degradation study is needed).

## 7. HFMC Modeling Road Map

**Figure 10:**
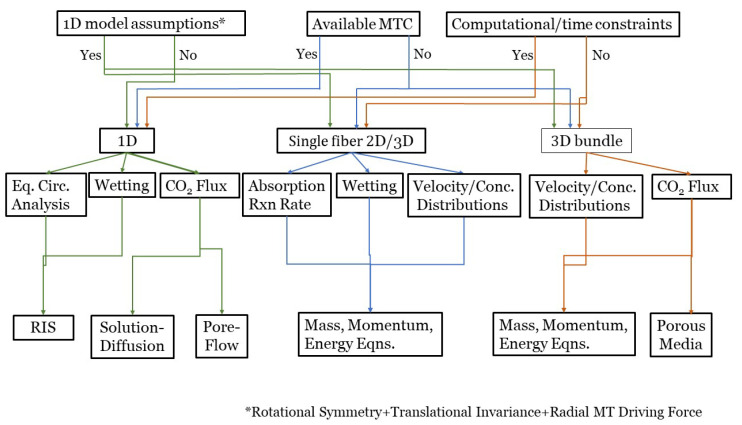
The road map is separated based on three defining questions: (1) Is the modeler taking into account 1D model assumptions? (2) Does the modeler have access to the mass transfer coefficient values? (3) Does the modeler have computational or time constraints? Depending on those qualifications, 1D, 2D, or 3D models can be chosen. Each modeling approach previously described for 1D, 2D, and 3D models provide specific end goal phenomena to be described. These goals for 1D models shown here are solving for the equivalent circuit analysis (eq. circ. analysis), the wetting effects, and CO${}_{2}$ flux removal. Depending on the overall goal, the RIS, solution-diffusion or pore flow model could be used. For 2D axisymmetric/3D single fiber models, the end goal could consist of observing the absorption reaction rate, the wetting effects and overall velocity and CO${}_{2}$ concentration profiles. The mass, momentum and energy equations could be coupled to recover the velocity and concentration profiles in all three domains (tube, membrane, and shell domains). Finally, 3D models observe the overall bundle of the HFMC. If the final goal is to determine detailed fluid and concentration distributions within the bundle, the mass, momentum and energy equations should be used for more accurate results. However, if the overall goal is to observe the CO${}_{2}$ flux rate, the porous media approach should work just as well.

This section summarizes the exposed ideas presented in Section 3, Section 4 and Section 5 to guide modelers’ decision towards the type of modeling approach and dimensionality they should choose for their research goals. A road map, Figure 10, illustrates possible directions the simulation could take.

## 8. Applications and Challenges

In the following paragraphs, we wish to highlight the application aspect of several groups’ HFMC post-carbon capture 1D, 2D, and 3D models. This section will also cover software implementation issues to model HFMCs and challenges associated with these simulations and models.

### 8.1. Applications of 1D Models

As previously stated, the RIS model can be used to determine the overall MTC needed to calculate the flux across the HFMC. The overall MTC can be modified to include chemical and wetting effects. For example, Boributh et al. [16] contributed to the field by creating a mathematical model to predict the physical absorption of CO${}_{2}$ and the effects of membrane wetting on pore size, membrane geometry (thickness and fiber length) and fluid flow. The results were validated with the experimental data reported by Achariyawut et al. [84]. Building on their work, this group incorporated the chemical effects of the system to predict the absorption performance of CO${}_{2}$ from a gas mixture containing methane (CH${}_{4}$). Using MEA as the absorbent, the group successfully predicted the performance of a PVDF HFMC by incorporating membrane wetting and the enhancement factor, *E*, to calculate the overall MTC and model the rate of absorption [18]. The RIS model was also used to predict CO${}_{2}$ separation from a CO${}_{2}$-N${}_{2}$ gaseous mixture using a DEA solution and a PP HFMC [26]. This group also incorporated partial membrane wetting into their model and observed a rapid decline in module performance due to the physical geometric changes, such as enlarged pore-size and elevated surface roughness. It was noted the pore-size enlarged quicker using DEA rather than MEA.

The solution-diffusion model is used to model dense membranes, such as dense polymer- supported ionic liquid membranes used to separate CO${}_{2}$-N${}_{2}$ and CO${}_{2}$-CH${}_{4}$ mixtures [93]. Models predicting the performance of HFMCs using the solution-diffusion approach have successfully investigated process intensification (for scale-up purposes) and solvent leak reduction when considering a volatile solvent, such as aqueous ammonia [99]. This work has been validated against experimental work in the laboratory scale [137]. Chabanon et al. [19] point out a challenge comparing the experimental results against this well-established model: it is unrealistic to compare the results without adjusting any parameter. Therefore, the membrane MTC is adjusted in the model for validation against the experimental results. The solution-diffusion model approach has also been used to model separation CO${}_{2}$ from multi-component flue gas containing N${}_{2}$, O${}_{2}$, H${}_{2}$O, and CO${}_{2}$ to find optimal regions of flue gas pressures and membrane area [91].

### 8.2. Applications of 2D Models

Many research groups have applied 2D-axisymmetric HFMC models to post-combustion carbon capture. Many different solvents, operating conditions, flue gas mixtures, and wetting effects have been modeled in this framework. Many of these models have successfully predicted experimental results for PCC applications.

For example, Shirazian et al. [43] developed a 2D-axisymmetric model to study CO${}_{2}$ removal from 20/80 CO${}_{2}$-N${}_{2}$ mixture for general gas separation (coal, natural gas and flue gas). They initially only studied physical absorption to isolate the effects of varying temperature and fluid and gas flow rates. They then studied the impact of chemical absorption by incorporating MDEA [31], diethanolamine [60], DEA [61], MEA, 2-amino-2-methyl-1-propanol (AMP), and potassium carbonate (K${}_{2}$CO${}_{3}$) [23] into their models. Their results show that MEA achieved the highest CO${}_{2}$ removal rate. Another 2D modeling group studied the effect of mixing an ionic liquid into pure water to act as a physical absorbent alongside MEA as a chemical absorbent [62]. Their results showed that including an ionic liquid increases CO${}_{2}$ absorption in both physical and chemical absorbents.

Other groups have modeled wetting effects in their 2D axisymmetrical models of HFMCs. Non-wetted, partially wetted, and fully wetted models were compared by one team to observe the effect membrane wetting has on separation efficiency, CO${}_{2}$ flux and overall MTC [63]. They successfully modeled the removal of CO${}_{2}$ from a 15/85 CO${}_{2}$-N${}_{2}$ gas mixture and observed with increasing membrane wetting, there was an increase of mass transfer resistance and therefore lower separation efficiency and CO${}_{2}$ flux. Abdolahi et al. [64] also modeled a 2D-axisymmetric HFMC with partial membrane wetting and found that even a 10% wetting of the membrane reduces the efficiency of the CO${}_{2}$ removal process by more than 47%. Their results were compared to experimental data.

Ghasem et al. [65] developed another 2D-axisymmetric HFMC model for the simultaneous absorption/stripping of CO${}_{2}$ with potassium glycinate. This group was able to use two gas-liquid HFMC in parallel and model both the absorption and stripping process. They were able to model the stripping portion by defining the reaction rate in the solvent-side as a function of temperature and concentration, observing that, as the stripping temperature of rich solvent increases, the stripping efficiency increases. Their work was validated against experimental data.

### 8.3. Applications of 3D Models

Very few 3D models currently exist for HFMCs in PCC applications. Thanks to recent advances in computational ability, this is an emerging research area with room for future development. One research group has developed a 3D bundle model to study the flow passing through different fiber array arrangements for CO${}_{2}$ removal from CH${}_{4}$[121]. Three-dimensional modeling is needed for this group’s work to study the radial cross-flow distribution for inline and staggered fibers in the bundle, as well as momentum mixing. They demonstrated that a bundle with staggered arrangement outperforms the bundle with the inline arrangement after evaluating the CO${}_{2}$ flux rate across the membrane surface. The porous media approach, while scarce in the PCC field, has recently been applied by Pozzobon et al. [136]. This group created a computational fluid dynamic model that describes the mass transfer at the fiber scale in addition to the fluid flow in the bundle. The purpose of this work is to illustrate how to apply the porous media model to obtain the mass transfer resistance values numerically, rather than using a correlation, like for 1D models, or experimental values, like for 2D models. Their results replicated Whitaker [138] and Fougerit et al. [139,140] correlation’s in addition to experimental runs.

### 8.4. Software Implementations

One of the key challenges with developing HFMC models for PCC is determining whether a 1D, 2D, or 3D model is the best option. The decision to model a HFMC in 1D, 2D, or 3D depends not only on governing assumptions and desired results but also on practical computing constraints. Carbon capture HFMC models require a complex combination of equations that may be linear, non-linear, possibly coupled, ordinary-differential or partial-differential. Therefore, sophisticated modeling software (e.g., MATLAB ${}^{®}$, COMSOL Multiphysics${}^{®}$, ANSYS (CFX and Fluent), ASPEN Custom Modeler, OpenFOAM) are often used. Software selection depends on the complexity of the model and what features need to be studied. For example, MATLAB${}^{®}$ is typically sufficient for 1D modeling, but finite-element software products (e.g., ANSYS, COMSOL Multiphysics${}^{®}$) are often preferred for 2D or 3D modeling as they do not require coding. ANSYS and COMSOL both have user-defined partial differential equations (PDEs) and pre-defined PDEs modules for a wide range of applications, putting less burden on the user during initial model set-up. Although ANSYS and COMSOL Multiphysics${}^{®}$ are used for 1D models, as well, their true advantage is amply revealed in the 2D and 3D simulations where complexity increases dramatically [128]. MATLAB${}^{®}$ is a popular platform for 1D and sometimes 2D HFMC models [103,141]. Some 1D models can even be set up in Excel workbooks or similar platforms. Researchers may opt for 1D models based on cost constraints and software availability. ASPEN Custom Modeler incorporates HFMC models as a user defined unit operation to study scale-up modeling [142].

Mesh refinement is required in narrower areas of the geometry demanding higher resolution to guide the computations. For example, the membrane domain in the 2D single fiber analysis will need finer mesh to fully capture the physics that occur within a thinner domain. This is especially true when the model needs to capture membrane wetting effects. Another example where mesh refinement is needed is for a 3D model of the individual fibers to capture the physics within the smaller gaps between the fibers [102]. However, mesh refinement will cause the simulation to become more computationally expensive. Therefore, the modeler needs to determine the most demanding physics of the system using non-dimensionalization and the knowledge of boundary layers to assess the needed mesh sizes. Mesh independence is another important factor of the meshing methodology, used to determine accurate finite element/volume solutions. It is highly recommended for 2D and 3D simulations of HFMCs. The mesh independence study chosen will provide a less burdensome model that solves the physics to the desired level of accuracy. Once the simulation produces minimal changes between the mesh element or volume solutions for different levels of mesh refinement, the less burdensome mesh will be chosen and the mesh independence study will be complete. In general, models should use the least number of mesh elements necessary to converge on a satisfyingly accurate solution. Examples of authors that have published their mesh study for HFMCs can be found here [64,121].

One of the main downsides of 3D HFMC modeling is that it is computationally expensive, which may prevent some researchers from pursuing this route. However, sophisticated multiphysics software packages (e.g., ANSYS, COMSOL Multiphysics${}^{®}$) facilitate 3D modeling of HFMCs. These user-friendly software packages also have built-in short-cuts for reducing computational time (e.g., reducing mesh size, simulating a symmetric portion of the full geometry). Three-dimensional simulations of HFMCs can also often be run in parallel on different machines in a computer cluster to minimize computational time. Recent advances in software and computers have made it possible to perform 3D simulations of HFMCs faster and cheaper. Future advancements in computation will enable more researchers to pursue complex 3D models for carbon capture HFMCs.

### 8.5. Modeling Challenges

One shortcoming of current HFMC models for PCC is that they are exist no models capturing physical and chemical degradation of the membrane over time. Degradation rates are typically characterized experimentally, but these experiments are expensive and lengthy. Transient models that can predict long-term degradation of HFMCs exposed to flue gas would be immensely helpful. Although it would be theoretically and computationally challenging to develop a detailed mechanistic model for long-term degradation, simple models could be developed to extrapolate short-term experimental degradation data to longer time scales. This kind of model could save time and money on pilot testing and enable quicker scale-up of HFMC technology.

Another challenge that HFMC models face is that they struggle to capture non-idealities in bundle design. Because most HFMC models assume a uniform distribution of fibers with identical stream conditions, they cannot predict the effects of flow maldistribution, non-uniform fibers, or uneven fiber distribution within a bundle. For example, Happel’s free surface model (which is used in 2D models to determine the shell-side velocity) assumes that all the fibers are evenly spaced in a triangular or staggered array. The actual shell-side velocity could vary substantially from this model’s predictions, if fibers are distributed unevenly. Similarly, most HFMC models assume no friction from the walls of the fiber. Three-dimensional HFMC models can address many of these short-comings by incorporating wall effects and bundle non-uniformities.

Two other assumptions included in most models that could be considered as rendering the models prone to wrongful representation of the physics are: (a) constant permeate and retentate mixture properties, i.e., not function of the local concentration, and (b) continuity equation described by incompressible flow, i.e., ∇u=0. The latter assumption is posed to apply the continuity equation to solve for the velocity in the tube-side. Both these hypotheses generate concern, since the gaseous system is a multi-component flue gas mixture. Therefore, the density will change as CO${}_{2}$ is separated. This is an issue that needs to be addressed with most of the relevant reported research and remedied in future modeling undertakings to produce more reliable results.

## 9. Scale-Up Modeling from Lab Scale to Commercial Scale

The previous sections have focused exclusively on modeling a single fiber or bundle of fibers in a carbon capture HFMC absorber. However, modeling efforts need to extend beyond small-scale HFMC absorbers in order to scale up work from the lab to the pilot and commercial scales. This would help minimize the transitional time between bench and large scale HFMCs absorbers and minimize the risk and costs for the plant facilities. Scale-up and commercialization efforts tend to increasingly rely on modeling, as necessary stepping stone to design and optimize scale-up for PCC. A characteristic example is the Carbon Capture Simulation for Industry Impact ($CCSI^{2}$) program of the National Energy Technology Laboratory (NETL)-Department of Energy (DoE). $CCSI^{2}$ is a computational tool-set of different scale and scope models, ranging from CFD and process modeling to optimization, uncertainty quantification and sequential design of experiments, targeted to de-risk and facilitate up-scaling PCC technology in the United States. Apart from the standard CFD and process models, new capabilities pertaining to Artificial Intelligence are added to the researchers’ portfolio. For example, modeling efforts involving systematic design of experiments (referred to as sequential design of experiments) can guide test campaigns and enable teams to get experimental results efficiently. Techno-economic models of carbon capture HFMC systems can also aid with the scale-up process by predicting and optimizing for parameters like size and cost. Some HFMC scale-up models focus exclusively on optimizing the HFMC absorber design (e.g., to minimize size or cost) at a larger scale. Other HFMC scale-up models include the entire carbon capture system or even the whole power plant system. Aspen Custom Modeler and similar software packages are helpful tools for developing and optimizing process models for complex systems like this.

**Figure 11:**
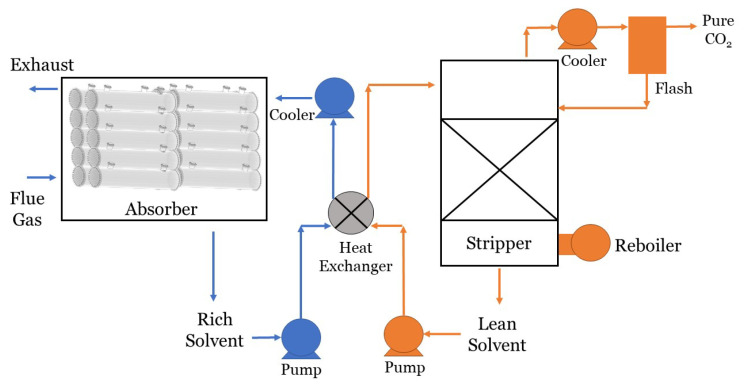
Graphic of a conventional CO${}_{2}$ capture process using HFMC modules in the absorber. The blue represents the absorption process, and the orange represents the stripped portion of the system.

Figure 11 shows a conventional CO${}_{2}$ capture process using HFMC modules as the absorber. This CO${}_{2}$ capture system also requires a CO${}_{2}$ stripper, coolers, pumps, and a reboiler.

Although there have been several pilot tests of carbon capture HFMCs [88,143,144], modeling efforts for gas-liquid HFMC pilot-scale or commercial-scale modeling are scarce [44]. The following discussion therefore focuses on gas-gas HFMC carbon capture systems, which closely resemble gas-liquid carbon capture HFMC systems. A common metric used to quantify CO${}_{2}$ capture cost in membrane systems is the gas processing cost. The gas processing cost depends on various parameters (e.g., membrane geometry, operating conditions), so a final product purity must be specified in order to achieve a desired gas processing cost goal [142]. The overall calculation of gas processing cost depends on the capital related costs for installation and fabrication of equipment, the variable operating and maintenance costs, and the cost of hydrocarbons lost in the permeate stream [145]. In most instances, researchers also consider a payout period of 5 years to calculate the total capital cost. Researchers often use gas processing cost to study the impact of non-ideal cases that could occur in industrial applications. For example, variable permeances due to temperature and pressure dependence greatly affect the membrane permeability [146], and membrane fouling increases the energy requirement [147]. In addition, up to 20% of the base plant costs should be allocated to cover unforeseen events [148], such as complications with the recycle stream [149,150]. The same gas processing cost methodology is used across different projects to study the various parameters that affect gas processing cost, such as pressure ratios [151] and flow pattern distributions [152].

Aside from gas processing cost analyses for gas-gas HFM modeling, many researchers shift their modeling efforts from modeling single fibers to modeling pilot-scale membrane systems when it is time to scale up their technology. ASPEN HYSYS${}^{®}$ is a commercial process simulation program that can be used for this task, particularly if the team wishes to integrate their membrane model into a process model for an entire power plant. Modeling in this framework elucidates many bulk effects, such as non-uniform distribution of flow among fibers, that have been used for HFMCs but are difficult to capture in smaller-scale HFMC models [153]. Proprietary platforms, like the Integrated Environmental Control Model (IECM) from Carnegie Mellon University, estimate system and performance costs of power plants with gas-gas membrane carbon capture [154]. In most of these process models, researchers consider a two-stage membrane process: the first membrane operates at an optimal pressure ratio [155], and the second membrane focuses on selectivity [156]. Platforms, like IECM, could likely be used to model gas-liquid HFMCs, as well. This would enable researchers to determine the optimal process design for gas-liquid carbon capture HFMC systems.

System modeling is a crucial step for carbon capture HFMC technology development. Particular emphasis should be placed on characterizing the CO${}_{2}$ stripping process, which is often overlooked in carbon capture HFMC modeling studies. The most expensive operating cost in a solvent-based CO${}_{2}$ capture system is typically the cost of desorbing CO${}_{2}$ from the solvent [157]. Therefore, more effort is needed to model desorption and optimize solvent selection in HFMC systems. These models could complement experimental efforts to minimize costs of HFMC carbon capture systems and make them more competitive with traditional solvent carbon capture systems.

## 10. Conclusions and Recommended Future Work

HFMCs are one of the leading technologies for post-combustion carbon capture. Modeling efforts are needed both to characterize these HFMC technologies and scale them up for commercial adoption. This review paper presented and compared 1D, 2D, and 3D modeling approaches for carbon capture HFMCs. The goal of this review is to help HFMC researchers identify which modeling methods are most applicable to their projects. One-dimensional models are the most efficient and tend to produce accurate results given the correct assumptions (such as treating the interfaces of each domain as ”films” for the RIS model). Modeling in 2D has also become a popular option in carbon capture HFMC research, and it provides higher accuracy and more information than 1D models can provide. One-dimensional and 2D models for membrane wetting were also discussed.

Three-dimensional models of carbon capture HFMCs are scarce because they are more computationally expensive than 1D or 2D models. However, they are qualitatively different in the nature of information they can reveal. Three-dimensional models are recommended for researchers that need to study variations within the HFMC bundle, which cannot be encompassed by 1D or 2D single fiber models. Similarly, if what is of interest is understanding of the underlying mechanisms of mass, energy, and momentum transfer and their interplay, then one has to resort to 3D bundle models or, at the very least, to 2D and 3D single fiber models with some effort to account for non-idealities from up-scaling to the bundle level. For those interested in capturing bundle variations with reduced computational cost, we recommend considering the porous media modeling approach. Further research is also needed to accurately model membrane fouling. These efforts will help researchers better predict HFMC lifespan and minimize degradation.

The transition from bench-scale to pilot-scale modeling was also discussed, and it is an area where further research is needed in order to make HFMCs competitive with other CO${}_{2}$ separation technologies [158]. While scale-up models exist for gas-gas HFMCs, more scale-up models are needed, specifically for gas-liquid carbon capture HFMCs. Future research on both scale-up modeling and 3D bundle modeling will accelerate the progress and commercialization of gas-liquid carbon capture HFMCs.

## Figures and Tables

**Table 1:** Summary of common solvents used in hollow fiber membrane contactors (HFMCs) for CO${}_{2}$ separation from flue gas.

Water (H${}_{2}$O)	[14,15,16,17]
Monoethanolamine (MEA)	[15,17,18,19,20,21,22,23,24]
Diethanolamine (DEA)	[24,25,26,27]
Sodium Hydroxide (NaOH)	[28,29]
Methyldiethanolamine (MDEA)	[30,31,32]

**Table 2:** Summary of common membrane materials used in hollow fiber membrane contactors (HFMCs) for CO${}_{2}$ separation from flue gas.

Polypropylene (PP)	[14,22,25,26,33]
Polytetrafluoroethylene (PTFE)	[15,21,34]
Polyvinylidene fluoride (PVDF)	[19,22,25,29,35,36,37]
Polymethylpentene (PMP)	[19]

**Table 3:** Concentration boundary conditions in a 2D axisymmetric HFMC with solvent flowing into the tube side at z=L and flue gas flowing into the shell side at z=0, as shown in Figure 7 [60].

Position	Tube	Membrane	Shell
z = 0	$C_{CO_{2}-tube}=0$, $C_{solvent}=C_{solvent-inlet}$		
z = L			$C_{CO_{2}-shell}=C_{CO_{2}-inlet}$
r = 0	$\frac{\partial C_{tube}}{\partial r}=0$		
r = R${}_{tube}$	$C_{CO_{2}-tube}=C_{CO_{2}-membrane}\cdot m$, $\frac{\partial C_{solvent}}{\partial r}=0$	$C_{CO_{2}-membrane}=\frac{C_{CO_{2}-tube}}{m}$	
r = R${}_{membrane}$		$C_{CO_{2}-membrane}=C_{CO_{2}-shell}$	$C_{CO_{2}-shell}=C_{CO_{2}-membrane}$
r = R${}_{shell}$			$\frac{\partial C_{CO_{2}-shell}}{\partial r}=0$

**Table 4:** Boundary conditions for the two additional mass transfer equations that account for a partially wetted membrane in a 2D axisymmetric HFMC model [7].

Position	Gas-Membrane	Liquid-Membrane
r = R${}_{tube}$		$C_{CO_{2}-l-membrane}=C_{CO_{2}-l}$
r = R${}_{membrane}$	$C_{CO_{2}-g-membrane}=\frac{C_{CO_{2}-l-membrane}}{m}$	$C_{CO_{2}-l-membrane}=C_{CO_{2}-g-membrane}m$, $\frac{\partial C_{CO_{2}-membrane}}{\partial r}=0$
r = R${}_{shell}$	$C_{CO_{2}-g-membrane}=C_{CO_{2}-shell}$	

**Table 5:** State-of-the-art HFMC models for PCC organized by dimensionality. Modeling assumptions (listed in the text) are checked for each model to enable comparison.

Dimension	References	Assumptions															
		1	2	3	4	5	6	7	8	9	10	11	12	13	14	15	16
1D	Boributh et al. [16]	✓	✓					✓								✓	✓
	Zaidiza et al. [135]	✓	✓	✓	✓	✓										✓	✓
	Khaisri et al. [21]	✓	✓				✓									✓	✓
	Boributh et al. [68]	✓	✓			✓		✓								✓	✓
	Boributh et al. [18]	✓	✓			✓		✓								✓	✓
	Rode et al. [73]	✓	✓						✓							✓	✓
	Zaidiza et al. [97]	✓	✓		✓	✓										✓	✓
	Villeneuve et al. [99]	✓	✓	✓	✓	✓			✓							✓	✓
	Li et al. [82]	✓	✓		✓				✓							✓	✓
	Chu et al. [96]	✓	✓			✓										✓	✓
	Haddadi et al. [102]	✓	✓			✓				✓		✓				✓	✓
	Cui et al. [51]	✓	✓				✓		✓		✓	✓			✓	✓	✓
	Saeed et al. [72]	✓	✓		✓		✓									✓	✓
2D	Al et al. [14]	✓	✓				✓			✓	✓	✓	✓	✓	✓	✓	✓
	Shirazian et al. [43]	✓	✓				✓			✓	✓	✓	✓	✓	✓	✓	✓
	Rezakazemi et al. [30]	✓	✓				✓			✓	✓	✓	✓	✓	✓	✓	✓
	Shirazian et al. [31]	✓	✓				✓			✓	✓	✓	✓	✓	✓	✓	✓
	Shirazian et al. [60]	✓	✓				✓			✓	✓	✓	✓	✓	✓	✓	✓
	Hosseinzadeh et al. [114]	✓	✓				✓			✓	✓	✓	✓	✓	✓	✓	✓
	Faiz et al. [15]	✓	✓				✓				✓		✓	✓	✓	✓	✓
	Ghasem et al. [111]	✓	✓				✓					✓	✓		✓	✓	✓
	Goyal et al. [26]	✓	✓							✓	✓					✓	✓
	Li et al. [85]	✓	✓	✓						✓		✓				✓	✓
	Cao et al. [109]	✓	✓							✓	✓	✓				✓	✓
	Shirazian et al. [110]	✓	✓							✓	✓	✓			✓	✓	✓
	Nakhjiri et al. [112]	✓	✓							✓	✓	✓			✓	✓	✓
	Qazi et al. [63]	✓	✓							✓	✓	✓				✓	✓
	Abdolahi et al. [64]	✓	✓							✓	✓	✓				✓	✓
	Ghasem et al. [65]	✓	✓							✓	✓	✓			✓	✓	✓
	Qazi et al. [113]	✓	✓							✓	✓	✓			✓	✓	✓
3D	Boucif et al. [119]	✓	✓				✓			✓	✓		✓		✓	✓	✓
	Boucif et al. [120]	✓	✓	✓	✓		✓						✓			✓	✓
	Usta et al. [121]	✓	✓				✓									✓	✓
	Cai et al. [133]	✓	✓				✓				✓					✓	✓
	Pozzobon et al. [136]	✓	✓				✓				✓					✓	✓

## Nomenclature

**Subscripts**	
l	liquid
m	membrane
g	gas
ov	overall
eff	effective
i	inner
o	outer
h	hydraulic
w	liquid pore
f	membrane pore
max	maximum
r	radial coordinate
CO${}_{2}$	CO${}_{2}$ in a domain
θ	tangential
z	axial coordinate
avg	average
A	component
rxn	reaction
t	time
i	domain
th	thermal
j	species
**Variables**	
k	resistance
D	diffusion coefficient
ε	porosity
τ	tortuosity
δ	thickness
d	diameter
*E*	enhancement factor
L	length
C	concentration
R	fixed radius
r${}_{A}$	reaction rate constant
φ	volume fraction
γ	surface tension
m	solubility of CO${}_{2}$
V	volume
γ	surface tension
$\kappa_{D}$	Darcy permeability
$\kappa_{1}$	inertial permeability
$x^{*}$	wetting ratio
v	velocity vector
$\dot{v}$	volumetric flow rate
η	effective capture ratio
*a*	interfacial area
*H*	Henry’s constant
*P*	partial pressure
*B*	pore geometry coefficient
*T*	temperature
θ	contact angle
*n*	number of fibers
ρ	density
μ	viscosity
g	gravity
$\dot{n}$	molar flow rate
*∂*	partial differential
*▵*	difference
b	body force vector
L	gradient of velocity
*f*	constitutive parameter
**Acronyms**	
HFM	Hollow fiber membrane
HFMC	HFM contactor
CC	Carbon capture
PCC	Post-combustion carbon capture
RIS	Resistance in series
MTC	Mass transfer coefficient
CFD	Computational Fluid Dynamics
